# Endovascular Treatment of Vertebral Column Metastases Using Intra-Arterial Cisplatin: Pilot Experience

**DOI:** 10.1155/2014/915904

**Published:** 2014-05-22

**Authors:** Bohdan W. Chopko

**Affiliations:** Department of Neurosurgery, Stanford University Medical Center, 300 Pasteur Drive, Stanford, CA 94305-5327, USA

## Abstract

*Background and Importance*. Treatment of spinal column metastatic tumors is challenging, especially in the setting of progressive disease despite previous radiation and chemotherapy. Intra-arterial chemotherapy is an uncommonly used but established treatment for head and neck cancers, retinoblastoma, and glioblastoma. The author reports extension of the IAC concept to vertebral metastatic tumors. *Clinical Presentation*. Two patients with intractable spinal pain secondary to spinal metastatic involvement at T11-L1 segments were treated with intra-arterial injections of cisplatin, with simultaneous sodium thiosulfate chelation. The first patient, a 60-year old female with metastatic lung carcinoma underwent, three cycles of therapy over a 9-week period; the treated regions demonstrated bone remodeling and sclerosis. The second case was a 40-year old male with malignant pheochromocytoma, who underwent a single treatment and succumbed 5 weeks later from progressive widespread disease. Both patients reported significant pain relief and neither of them exhibited a decline in neurologic function. *Conclusion*. The intra-arterial delivery of cisplatin appeared to be well tolerated in the two cases. In the case with the longest survival, the treated vertebral segments became more sclerotic, consistent with biomechanical stabilization. Endovascular treatment of spinal metastases may hold promise, especially as newer categories of biologic agents become more widely available.

## 1. Background and Importance

Intra-arterial chemotherapy (IAC) is a well-known but uncommonly utilized technique for treatment of solid visceral tumors, such as hepatocellular carcinoma, as well as sarcomas [1, 2]. In interventional neuroradiology, the IAC approach has been described for head and neck cancers as well as malignant glioma and retinoblastoma [3–5]. Advantages of IAC include intratumoral dose levels up to 250 times that of systemically administered intravenous methods, as well as the lack of first-pass inactivation prior to arrival at the tumor site [6]. Furthermore, in the case of cisplatin, simultaneous intravenous administration of sodium thiosulfate inactivates any cisplatin that has transited through the tumor bed, which greatly reduces the systemic toxicity.

In the case of spinal metastases, patients commonly receive radiation early in the treatment course, which subsequently precludes additional radiation and restricts treatment choices. Spinal column embolization is a well-described technique for decreasing blood flow to highly vascular lesions, such as arteriovenous malformations and neoplasms. By combining the endovascular access techniques of embolization with IAC, additional direct treatment of spinal column tumors can also be approached despite prior maximal radiation.

## 2. Clinical Presentation

Patients were considered a candidate for spinal IAC if they met the following criteria: (1) progression of spinal column tumor growth despite maximal spinal irradiation dosage and systemic chemotherapy; (2) disabling pain due to tumor progression, requiring escalating narcotic dosages; (3) absence of significant neurologic compromise due to tumor compression of neural elements; (4) angiographically accessible tumor feeder vessels. In the evening before the planned IAC, the patient was admitted and intravenously hydrated. During the administration of IAC, simultaneous intravenous administration of sodium thiosulfate at 9 grams/meter squared was delivered as a rapid bolus over a five-minute infusion time, followed by a maintenance infusion of 12 grams/meter squared in 1 liter of sterile water at 167 mL/hour. Once complete, hydration with intravenous 5% dextrose one half normal saline solution was administered at 125 mL/hour until discharge the following morning.

### 2.1. Case  1

**Figure 1:**
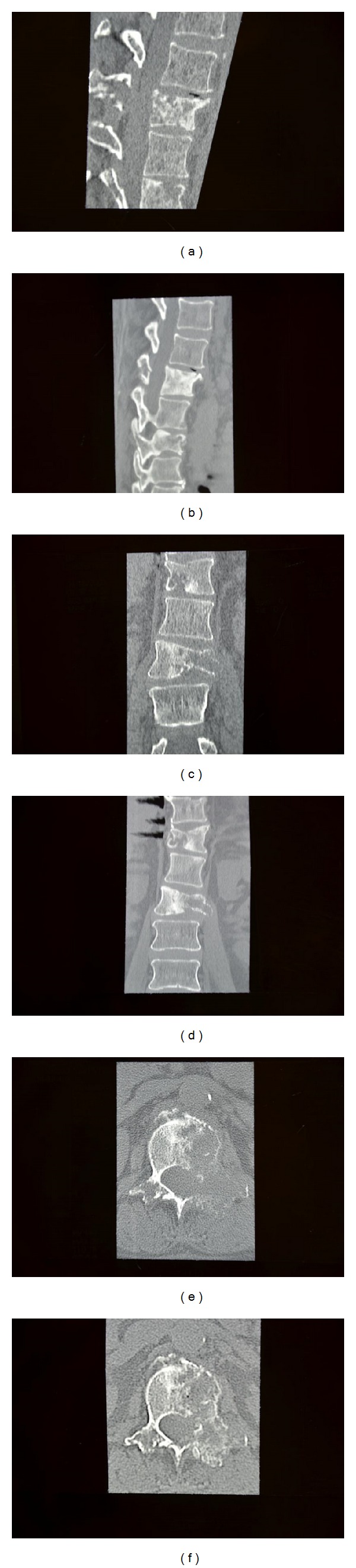
Case  1—computed tomography of thoracolumbar spine, noncontrast, demonstrating the development of bone remodeling and sclerosis 11 weeks after the initiation and 2 weeks after the completion of IAC (the vertebral body with maximal compression is L1). Sagittal before (a) and after (b), coronal before (c) and after (d), and axial L1 level before (e) and after (f) therapy.

A 60-year-old female with biopsy proven lung adenocarcinoma metastatic to T11, T12, and L1 vertebrae presented with a chief complaint of intractable axial thoracolumbar pain, which left her bedridden (Figure 1). She had previously received conventional intravenous chemotherapy and maximal spinal irradiation (3,500 centiGray) to the region. Power was graded at 4/5 in the bilateral lower extremities, with preserved sphincter function.

**Figure 2:**
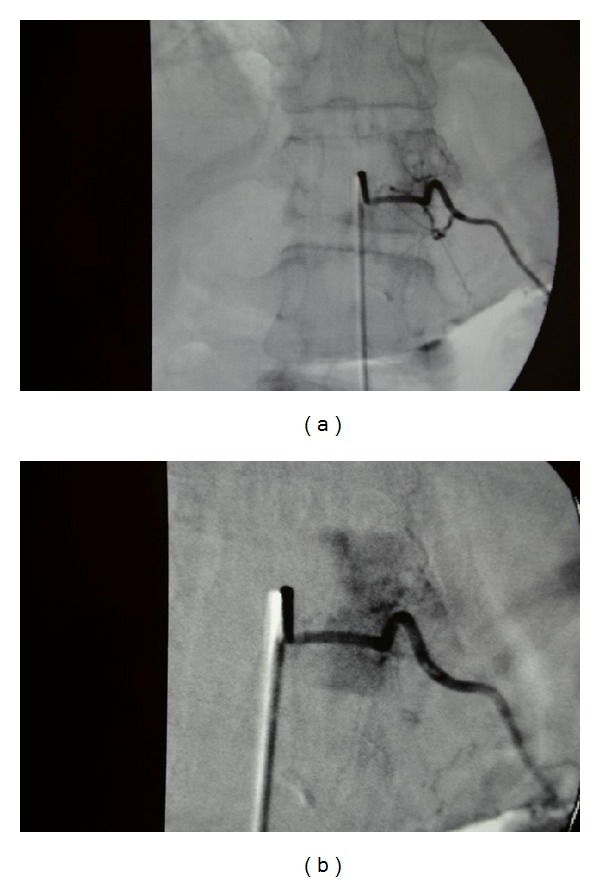
Case  1—unsubtracted (a) and subtracted (b) selective spinal angiogram performed within the left T11 spinal artery, anterior-posterior view, demonstrating position of catheter during treatment. Note the intense tumor blush involving most of the left hemivertebrae.

Over 9 weeks, she underwent three separate IAC cycles at approximate 2-week intervals with cisplatin (Figure 2). Cisplatin (1 mg/cc) was hand-injected into major tumor arterial feeders spanning T11 through L1 levels using 5 French diameters Cook HS-2 catheter (Bloomington, Indiana). The average dose per spinal arterial feeder was 74 mg, and the total average dose per session was 320 mg. Between 5 and 7 tumor feeder vessels were injected per session.

Durable pain reduction was experienced over the 14 weeks of therapy. Motor and sensory function of the legs and bowel and bladder function were preserved. She expired 6 weeks after the final IAC session from a massive thromboembolic stroke.

### 2.2. Case  2

**Figure 3:**
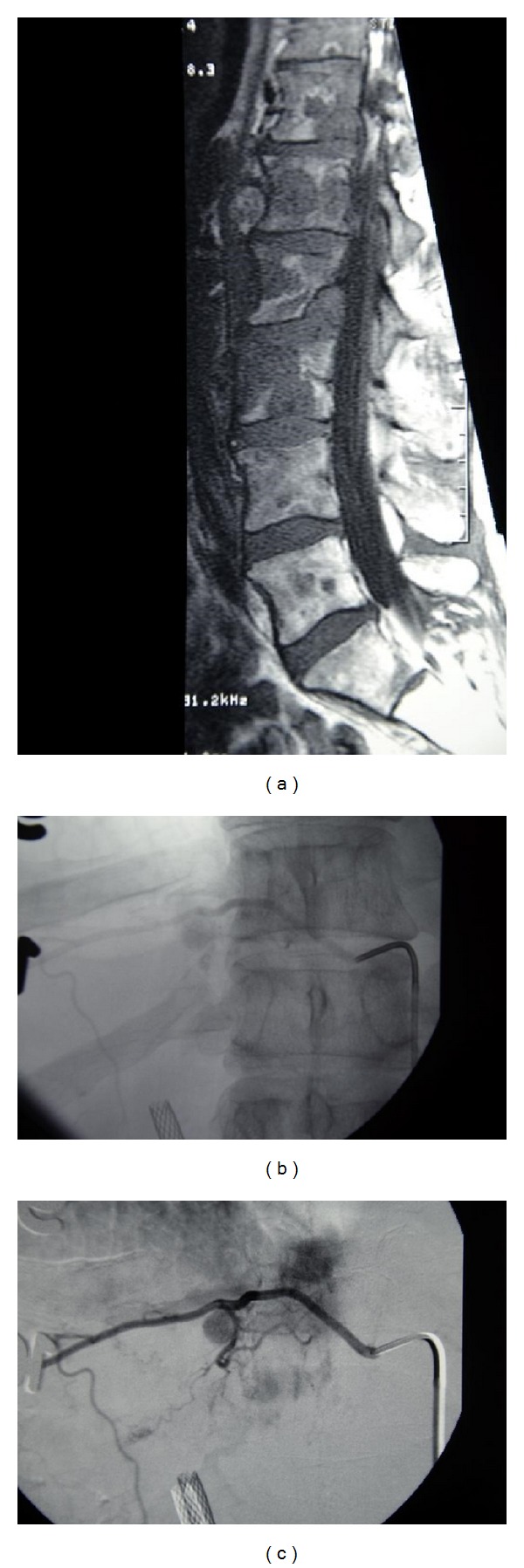
Case  2—widespread involvement of metastatic pheochromocytoma as seen on noncontrast T1 magnetic resonance sagittal image (a). Unsubtracted (b) and subtracted (c) anterior-posterior superselective angiographic run immediately prior to infusion of cisplatin into the left T11-T12 spinal artery. Note the extensive tumor blush, neovascularity, and paraspinal tumor nodule. A hepatobiliary stent is also present.

A 40-year-old male with a 5-year history of malignant pheochromocytoma presented with significant axial spinal pain and complete preservation of motor function of the extremities. Tumor was widely disseminated, including metastases to the entire axial spine, skull base, and periaortic and visceral lymph nodes (Figure 3). Ileus led to significant constipation, but urinary continence was preserved. Pain was centered chiefly at the thoracolumbar junction and persisted despite regional irradiation with 3,500 centigray.

He underwent a single treatment cycle of cisplatin (1 mg/cc) to the T11, T12, and L1 vertebrae. Cisplatin was hand-injected into the major tumor feeders using a Cook HS-2 catheter, 5 French diameters. A 200 mg total dose of cisplatin was administered, evenly distributed between 5 distinct arterial feeders.

No change in motor or sensory function of the legs was noted over the three weeks immediately following the IAC. His pain remained stable to somewhat improved. Progressive renal failure, ileus, and hepatobiliary obstruction led to encephalopathy and coma by 5 weeks after IAC, and support was withdrawn at 6 weeks after IAC.

## 3. Discussion

The two case studies demonstrate feasibility of an endovascular strategy in the treatment of metastatic spinal column disease. As no immediate neurologic deficits were apparent, it can be argued that, at least in the short term, the spinal cord and associated peripheral nerves are tolerant of direct arterial exposure to cisplatin.

The literature surrounding endovascular treatment of spinal tumors is scant. While the technique of preoperative embolization as a method of limiting blood loss is well described, the endovascular delivery of localized chemotherapy has been rarely reported. A single case of intra-arterial cisplatin infusion for treatment of a giant cell tumor of the lumbosacral spine was reported in 1982 [7]. The tumor masses were noted to develop rim calcification within months of treatment completion, which was interpreted to be a favorable response. Lin et al. [8] reported on the treatment of 9 patients with giant cell tumors of the sacrum using intra-arterial cisplatin combined with partial embolization; little information was included as to neurologic outcomes, but overall the tumors either stabilized or decreased in size as well as accumulating calcifications. In 1979 Hellekant [9] discussed the use of intra-arterial mitomycin via bronchial artery administration in the treatment of bronchogenic carcinoma, where great emphasis was placed on the potential for devastating neurologic damage from spillover into spinal cord perfusion beds. Fortunately, none of the profound side effects detailed by Hellekant were observed in the current series.

## 4. Conclusion

This small pilot experience underscores the potential for the application of endovascular techniques to spinal metastatic disease. While being technically feasible, however, the IAC approach carries significant downsides. The strategy relies on a labor-intensive and technologically sophisticated methodology, which demands economic and quality of life costs. If pain control alone is the goal, then the rationale for IAC is weak, as pharmacotherapy or functional procedures can be utilized. If, however, the aim is tumor cellular death, reversal and prevention of neural compression, and perhaps biologic modification of the diseased vertebral segment (such as osteoinduction), then IAC deserves further attention. In case 1, the treated vertebral segments transformed from blastic to sclerotic lesions, which raises the possibility of biomechanical stabilization via new bone growth, which may have accounted for some of the patient's pain reliefs.

As genetically tailored therapies for malignancy become more realized, then the concept of localized delivery may become more appealing. Additionally, agents beyond simply antineoplastic drugs, such as bone stabilizing or antiangiogenesis compounds, can be explored via endovascular delivery. While still being largely conceptual at this time, IAC of spinal tumors has the potential for life extension and quality improvement.
